# A Mobile App Offering Distractions and Tips to Cope With Cigarette Craving: A Qualitative Study

**DOI:** 10.2196/mhealth.3209

**Published:** 2014-05-07

**Authors:** Bernd Ploderer, Wally Smith, Jon Pearce, Ron Borland

**Affiliations:** ^1^Department of Computing and Information SystemsThe University of MelbourneParkville, VictoriaAustralia; ^2^The Cancer Council VictoriaMelbourne, VictoriaAustralia

**Keywords:** smoking cessation, relapse prevention, quitting, mobile phone, distraction

## Abstract

**Background:**

Despite considerable effort, most smokers relapse within a few months after quitting due to cigarette craving. The widespread adoption of mobile phones presents new opportunities to provide support during attempts to quit.

**Objective:**

To design and pilot a mobile app "DistractMe" to enable quitters to access and share distractions and tips to cope with cigarette cravings.

**Methods:**

A qualitative study with 14 smokers who used DistractMe on their mobiles during the first weeks of their quit attempt. Based on interviews, diaries, and log data, we examined how the app supported quitting strategies.

**Results:**

Three distinct techniques of coping when using DistractMe were identified: diversion, avoidance, and displacement. We further identified three forms of engagement with tips for coping: preparation, fortification, and confrontation. Overall, strategies to prevent cravings and their effects (avoidance, displacement, preparation, and fortification) were more common than immediate coping strategies (diversion and confrontation). Tips for coping were more commonly used than distractions to cope with cravings, because they helped to fortify the quit attempt and provided opportunities to connect with other users of the application. However, distractions were important to attract new users and to facilitate content sharing.

**Conclusions:**

Based on the qualitative results, we recommend that mobile phone-based interventions focus on tips shared by peers and frequent content updates. Apps also require testing with larger groups of users to assess whether they can be self-sustaining.

## Introduction

### Background

Quitting smoking is difficult. Although more than 90% of smokers have tried to quit at least once, between 85% and 97% of all quit attempts fail within 1 year [1,2], depending on the population and the extent of help they receive. A key challenge is managing cravings for cigarettes, particularly in the first month after quitting [3]. During this time, smokers who have quit (quitters) frequently experience cravings, often triggered by particular times of the day, activities, places, emotional states, and the presence of other smokers. Although nicotine replacement products can reduce withdrawal symptoms, people must manage these situations and cope with cravings to prevent a lapse or relapse [4,5].

Relapse prevention programs focus on providing quitters with skills to identify factors and high-risk situations that may lead to a lapse [6,7], including simple action plans like the four Ds of quitting: delay, deep breathing, drink water, or do something else. Additionally, relapse prevention programs focus on anticipatory strategies to help quitters prevent cravings and restorative strategies to help them in the aftermath of cravings [8,9]. The delivery of such relapse prevention programs through Web-based services can be effective [10,11] and cost-effective for counseling services [11,12].

The widespread adoption of mobile phones offers new opportunities to help quitters cope. In principle, mobile phones offer support at any time and place, ensuring that resources for coping are available in the high-risk situations when quitters may be tempted to lapse [13]. Studies of mobile phone services for quitters have focused on the delivery of personalized advice through text messages [14-18], video messages [19], and mobile apps [20]. A recent review of commercially available mobile apps for quitting found that most focus on providing personalized information through functions like calendars and cost-saving calculators [21]. However, other possible uses of mobile apps in smoking cessation remain under-researched.

Two potential benefits of mobile apps that form the focus of this study are first, to provide a source of distractions from cravings in the form of interactive content such as games and websites; and second, to provide opportunities for social interaction among quitters to exchange support. To explore these, we developed and evaluated a mobile app called DistractMe that presented two distinct types of content for coping: distractions and quitting tips. Social interaction was supported through sharing items and comments on those items. Cognitive distractions have long been used in other health interventions, for example to help patients cope with pain [22]. In the context of smoking cessation, previous studies have shown that distractions, together with breathing exercises and food and drinks, are among the most commonly used techniques to prevent relapse [23]. Some researchers have noted that mobile phones might play an important role in providing distractions from cravings [23,24], both as a cognitive distraction (through engaging content) as well as a behavioral distraction (by keeping one’s fingers’ busy). In a rare study, Rodgers et al [25] included general interest messages (about sport, fashion, trivia) as well as advice and tips relevant to quitting in their text messaging intervention, which improved cessation outcomes. However, there remains a lack of understanding about the effects of such distractions and the strategies of coping with cravings they might support, including the social interactions around them.

Therefore, the aim of this study was to identify the various coping strategies enabled by the DistractMe app and to provide insights and recommendations for similar mobile phone-based interventions. The approach chosen was a detailed qualitative analysis of a small sample of real-life quit attempts using the app, rather than a summative evaluation of its longer-term effectiveness in quitting.

## Methods

### A Research Through Design Approach

The study followed a research through design approach, involving the making and deployment of a technological prototype to generate knowledge about how it is used in practice [26,27]. Following this approach, the analysis phase of technology design was extended and centered on users and other stakeholders. We conducted several rounds of workshops with technology designers, smokers, and smoking cessation counselors to generate design ideas. Mock-ups of emerging elements were produced and evaluated in interviews with smokers [28,29], leading eventually to the design of the DistractMe app. Also following a research through ,design approach, the app was evaluated in a naturalistic setting to understand whether and how people adopt and use it in practice. Here the emphasis is on how the technology is appropriated by its users into their particular situations rather than presuming that they will use it in a way prescribed by the design. The analysis was based on a small sample of participants to develop a rich understanding of how the content items of the DistractMe app and related social interaction were deployed by participants in coping with cravings during the first few weeks of quitting.

### DistractMe App Design

**Figure 1:**
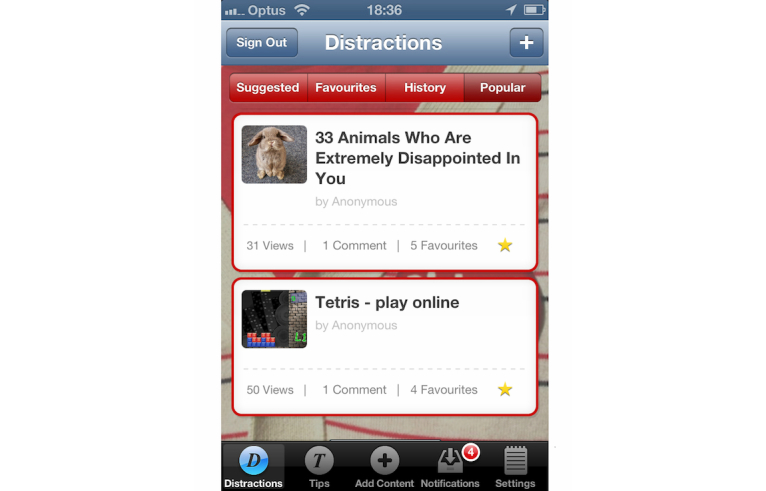
DistractMe includes a scrollable list of distractions. The red circle in the navigation bar highlights notifications of responses from other users.

**Figure 2:**
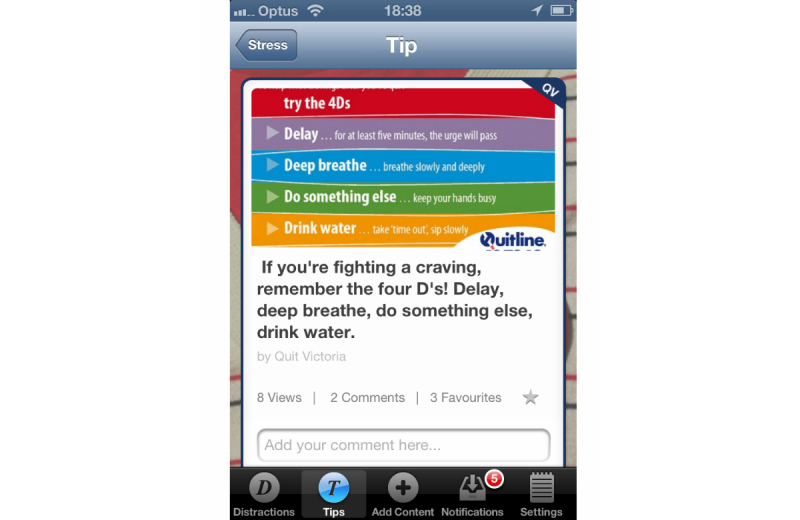
DistractMe provides tips to cope with cravings with the option to add a comment. The red circle in the navigation bar highlights notifications of responses from other users.

The essential idea of the DistractMe app was to provide quitters with convenient access to two kinds of content—distractions and tips—and to allow them to communicate with each other through comments on specific items. *Distractions* were nonsmoking related items that were intended to take people's minds off cravings and were typically links to interactive games and diverting Web-based content such as amusing images and videos. Users of DistractMe could access and filter a suite of digital content (videos, games, websites, images, etc) to distract themselves from craving as the need arose (Figure 1). *Tips,* in contrast, consisted of smoking-related information in the form of suggestions on how to cope with cravings and craving-inducing situations, such as “drink water during a craving” (Figure 2). Users could filter tips according to different types of situations, including feeling stressed or bored, or after they had eaten.

We envisioned different uses of distractions and tips. Distractions were to serve situations in which participants wanted to avoid thinking about smoking, whereas tips were for situations in which participants wanted to think about and fortify their quit attempt. DistractMe was intended for personal use to cope with cravings, but it also created possibilities for social interaction around the exchange of distractions and tips, and through shared comments on their effectiveness. Familiar social media functions were included in the app that allowed, for example, users to express “likes” and to see the number of views, comments, and favorites for each distraction (Figures 1 and 2). Furthermore, there was a notifications feature to highlight responses from other users and encourage contributions and comments. As discussed by Engeström [30] and as evidenced by popular social media such as YouTube and Reddit, images, videos, and short anecdotes are a popular means to encourage information sharing and social support among users.

DistractMe was implemented as an app rather than a Web service to ensure that it would be available to quitters at any time and place, even if they did not have mobile phone reception. The app was designed for iPhones because at the time of design, Apple’s IOS dominated the smartphone market with a share of 46% [31].

### DistractMe App Content Management

The content available on DistractMe came from both the research team and the participants in this study. The researchers played an active role in adding content to the app, because previous studies of online support groups have shown that the majority of users prefer to read rather than contribute content [32], and we did not aim to attract the large population of users needed to generate large volumes of content. At the outset, we prepared 171 distractions and 179 tips. Some tips were labeled as being sourced from the smoking cessation website of Quit Victoria (the organization of the fourth author) and others were based on cessation research and practice. They included suggestions for coping, motivational information, and success stories written by ex-smokers. Distractions were sourced on the Internet and edited with Quit Victoria’s smoking cessation counselors. They included interactive games and quizzes, funny pictures and videos, news, and infotainment websites. During the 6-week trial, the first author also added 37 comments on tips and distractions to engage with the study participants.

Participants could contribute distractions and tips directly through the iPhone app as well as through a Web form to simplify activities such as copy pasting or writing long texts. Each new item of content was immediately visible to the contributor, but required approval from a moderator to be visible to other users. The app was only available to users who consented to take part in the study.

### Study Participants

Participants were recruited through Quit Victoria’s telephone counseling service and their Facebook and Twitter channels as well as through the University of Melbourne’s staff and student mailing lists. We sought participants who owned an iPhone and would use the DistractMe app in the first weeks of their quit attempt because this is the period when relapse often occurs [3]. All participants had to have quit no more than 1 week before the first interview of the study or be planning to quit smoking in the next month.

In all, 27 participants were recruited but only 14 provided the data for this paper. Five were excluded because they did not make a quit attempt, 4 because they were not available for a second interview, and 4 because they did not use the app at all. The 14 participants who did make a genuine quit attempt were diverse in terms of age (ranging from 20 to 53 years, average 33 years), gender (11 female, 3 male), and nicotine dependence (ranging from very low dependence to high dependence according to the Fagerström test [33]). Success in quitting during the study was also variable. At the second interview 6-12 weeks into the study, 7 participants described themselves as nonsmokers, 2 as social smokers who had significantly reduced their smoking, and 5 as smoking at previous levels.

### Data Collection

Observations of quitting behavior with the DistractMe app were made over 6 weeks (Table 1). The study was timed to occur over the New Year holiday season in Australia because it is known that many people attempt to quit at this time. The first author interviewed each participant at the point of joining the study about plans to quit and general usage of iPhone apps (Multimedia Appendix 1). During this interview, participants downloaded and familiarized themselves with DistractMe. Interviews were conducted face-to-face (n=21) or via telephone (n=6) and lasted approximately 60 minutes. The participants received A$25 per interview to contribute toward travel expenses and phone data charges.

During the 6-week trial period, log data were collected for each participant. This included information about distractions and tips viewed or posted on the app. Participants were also asked to diarize their experiences with quitting and the app once a week. A second round of interviews was held after the 6-week trial focusing on the strategies and experiences of the quit attempts and the nature of engagement with the app (Multimedia Appendix 2). These lasted between 30 and 60 minutes, depending on the participant’s quit attempt. Both rounds of interviews were semistructured based on a short list of questions.

**Table 1:** Methods used during the 6-week field study.

Timeline	Method	Aims
Week 1	Interview 1	Discuss plans for quitting and gather first impressions with DistractMe app
6-12 weeks following recruitment	Log data	Collect information on contents viewed, posted, and commented on via DistractMe
By respondents over the period of use	Diary	Gather feedback on quit attempt and DistractMe at the time of quitting
6-12 weeks after starting, depending on when quit attempt was made	Interview 2	Reflect on quit attempt and the engagement with DistractMe

### Data Analysis

**Figure 3:**
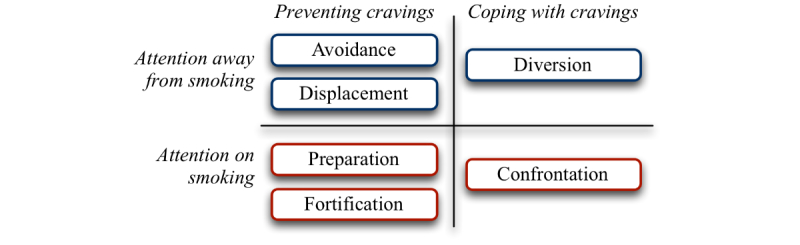
Six quitting strategies used in association with the DistractMe app.

The aim of the analysis was to develop an understanding of patterns of engagement with the DistractMe app, including its deployment in the quit attempt and the uses made of distractions, tips, and comments on them. As customary in many forms of qualitative research [34], the analysis was carried out in parallel with the collection of data. From the first interview onward, we wrote analytic memos for each participant that described instances of engagement with the app together with the researchers’ interpretations of the data [35]. These memos were used to code all forms of data (interview transcripts, diary entries, posts to the app) to develop an in-depth and broad picture of engagement with the app to cope with cravings and as a resource to later identify common themes across these instances [34]. The first author, using the software NVivo, conducted the coding and presented data and emerging themes in weekly meetings with the research team. Through ongoing discussions between all authors, observations were clustered into the 6 themes of engagement that are presented in this article [8]. As illustrated in Figure 3, themes were structured according to the content items of DistractMe (distractions and tips) and the stage of coping (anticipatory and immediate).

Qualitative analysis was complemented with data from the log files. These provided further evidence of the participants’ personal engagement with the app during their quit attempt, including when and how often users logged into DistractMe, what distractions and tips they viewed, and what content they added themselves. This provided a valuable cross-check on information provided in interviews. It was the basis of excluding nonusers and provided an indication of the level engagement among the active users.

## Results

### Overview

The log data indicated that the 14 active participants engaged more with tips than distractions. They consumed on average 22.1 tips (range, 0-68) and 16.4 distractions (range, 1-69). Whereas they posted more distractions than tips (mean, 2.3; range, 0-12 distractions, and mean, 1.5; range, 0-5 tips), they posted more comments on tips (mean, 1.6; range, 0-5) than on distractions (mean, 1.0; range, 0-5). These results confirmed the rich accounts given in interviews and diaries, which allowed us to develop a picture of the quitting strategies devised and deployed through use of DistractMe.

Participants reported a variety of episodes in which the distractions and tips exchanged through the app helped them to deal with cravings. As shown in Figure 3, we clustered these into 6 distinct quitting strategies associated with the use of DistractMe. Strategies were further organized along two dimensions: those for coping with cravings when they occur versus those for preventing cravings from happening or creating situations in which their effects would be diminished; and strategies that draw attention away from smoking versus those that focus attention directly on it.

There were 2 strategies to cope with cravings when they occurred: diversion, implying the distraction of attention away from the craving; and confrontation, implying a direct focus on the craving with the resolve to beat it. On preventing cravings, there were 4 distinguishable strategies. Two involved drawing attention away: avoidance of social or other situations in which temptations for smoking were high; and the displacement of cravings by embarking on other activities, such as gardening, that were less likely to lead to cravings or would diminish their effects. Two further strategies prevented craving by focusing directly on them: preparation, implying activities leading up to a quit date when supporting arrangements were put in place; and fortification, when quitters actively revisited their motivations and reaffirmed their commitment to quit. In the remainder of this section, we describe how DistractMe was appropriated by participants into these 6 strategies of quitting.

### Coping With Cravings

#### Diversion

Diversion was a strategy that was fully intended in the design of the DistractMe app. As described, the app provided potential diversion away from cigarettes toward content available on the mobile phone such as news articles, quizzes, and YouTube videos, as well as behavioral diversions through games that kept the fingers of the participants busy.

The following example illustrates how participant 2 used her mobile phone to seek diversion in this way. She had experienced a stressful situation at work, and back home in the evening she craved a cigarette to help her relax. However, instead, she used the DistractMe app in concert with other apps to divert herself.

I came home thinking, you know, I don’t know what to do with myself; I don’t know how to process all this. And after I’d sort of looked at a few newspapers online and had a bit of an interaction with some social media, I was sliding across the screen and went, Ooh, there we go, bingo! I logged on and all of a sudden 20 minutes later sort of went, Well, and the craving was gone. I didn’t even think about cigarettes for the rest of the night and felt a great deal more settled.

Participant 2, interview 2

At the outset of the trial, most participants were positive that distraction items could help in their quit attempt. In the second interview, however, although all of the 14 participants viewed distraction items for general interest, only 3 reported episodes where they had used them, or other content on their mobile phone, for diversion away from cravings. Many participants reported that DistractMe did not provide sufficient updates to effectively divert them.

There’s a lot of other applications that you can use and just like Pinterest. So if I want to just procrastinate or whatever I will use those applications because they have more content.

Participant 17, interview 2

This suggests that although diversion sounded appealing to the participants, possibly because it offered a simple strategy for quitting, in practice they generally preferred other strategies.

#### Confrontation

Confrontation was the mirror opposite of diversion. Rather than taking one’s mind off cravings for a cigarette, it consisted of concentrating on them to deliberately and actively resist. Confronting a craving appeared to be the more challenging response, and only 4 participants reported it.

The participants characterized confrontation as an inner dialogue in which they examined the craving and explained to themselves that it was only a withdrawal symptom rather than a physical need and that nothing adverse would happen to them if they did not respond. Participant 14, whose cravings were triggered by the stress of unexpectedly losing her home, described this inner dialogue in detail.

I was really stressed and I really wanted to buy a pack of cigarettes, and I knew it wasn’t going to make me feel better, but I just really wanted to do it. I had a little bit of a rant and a tantrum, and then I was just like—I just had to talk myself out of it. I was just like, You feel shit now, like smoking a packet of cigarettes is not going to make you feel better about the house, it’s not going to make you get the house anyway, like what difference does it make? It doesn’t. And I feel like I have to consciously talk myself out of those situations.

Participant 14, interview 2

The tips in DistractMe served as a means to support a confrontation strategy, which was consistent with our design intention for tips to be used to concentrate on smoking. For example, participant 17 used the videos in the tips section to remind herself of the negatives of smoking when she experienced cravings. Having experienced cancer in her family, she searched DistractMe for reminders about the health risks associated with smoking to withstand the temptation to give in and buy cigarettes.

If I’m stressed and if I feel like I have to run to the 7-Eleven [and buy cigarettes], I just feel like OK, let’s watch some creepy information about smoking on the app.

Participant 17, interview 2

DistractMe also supported confrontation by allowing quitters to share information that highlight that cravings are merely a withdrawal symptom that will not be solved by giving in. For example, participant 20 found a website that explained the physiological process behind cravings. Inspired by this website, she shared her suggestions to confront cravings through the DistractMe app.

Remind yourself, when you are experiencing a craving, your mind is sending messages that something bad will happen if you don't have a cigarette—messages similar to must eat or must drink for survival. When you are getting stressed about needing a cigarette, remind yourself that nothing bad will happen if you don't have a cigarette. The urgency, the craving itself, is a nicotine-induced fiction. And that uncomfortable desire will last about 3 minutes. Sit it out.

Participant 20, posted on DistractMe

### Prevention of Cravings

#### Preparation for Quitting

**Figure 4:**
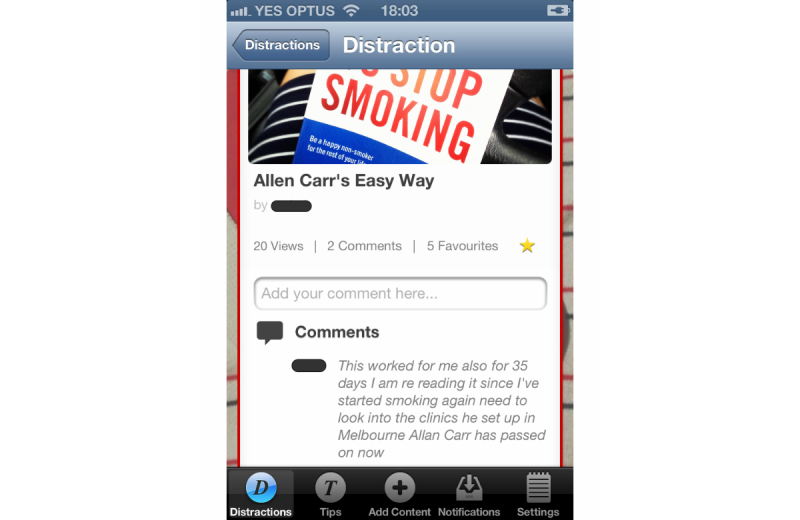
The participants used DistractMe to share resources to prepare for quitting.

Preparing for quitting took many forms. Virtually all participants prepared themselves mentally for their quit attempt by reflecting on experiences from previous quit attempts, working out a suitable time to quit, and looking up websites and reading books to get ready. For example, participant 14 posted a photo on the DistractMe app associated with the popular quitting guru Allen Carr as a motivator to stay a nonsmoker (Figure 4).

Only 6 of the 14 participants had set themselves an actual quit date at the time of the first interview. Three of these 6 people were planning to take quitting medication to reduce withdrawal symptoms, which required a prescription and so added a delay. The other 3 participants decided to quit in the days following the first interview and their preparation typically involved removing cigarettes from their environment.

Preparation is mainly just completely clearing the house of anything that reminds me of it, so everything I’ve used for smoking goes out.

Participant 20, interview 2

The remaining 8 participants were either continuously trying to quit or they were intending to quit in the near future and planned to quit ad-hoc, rather than on a chosen date.

Five participants reported that they used the DistractMe app for preparation before quitting. The most popular preparatory tip shared by the users of the app was to drink water, both to pre-empt cravings by staying hydrated as well as to cope with cravings by displacing the cigarette with a drink. Unlike other suggestions about medication and books, this tip could be applied instantaneously, and could continue after the quit date.

Well the drinking water tip—I started carrying a drink bottle with me everywhere, and I still do that, just always drinking lots of water.

Participant 24, interview 2

#### Fortification of the Quit Attempt

Eleven participants used DistractMe in their downtime to fortify their quit attempt. In particular, they used the tips to strengthen their motivation and dedication to stay a nonsmoker. Common strategies were to think about the negatives of smoking, highlight the positives for encouragement, and find inspiration and solidarity in reading about the experiences of others who were trying to quit.

For example, participant 7, a mother of 3, responded strongly to a video that showed how children would suffer if their parents were not there for them any more due a smoking-related premature death:

This ad really made me think how my three little ones would feel if I wasn’t around and all because of such a silly habit. Time to quit!

Participant 7, interview 2

**Figure 5:**
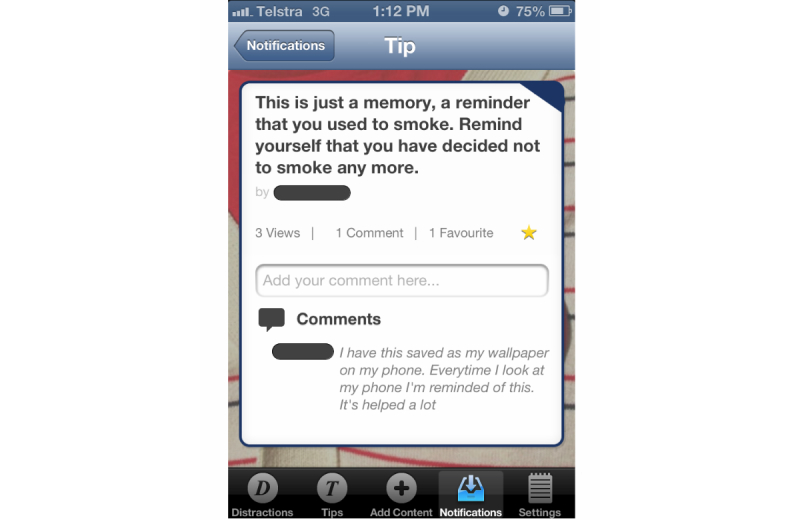
Participant 11 commented that she used this tip as a reminder to remain a nonsmoker as wallpaper on her phone.

Participant 11 used a screenshot of one of the tips as a background image on her phone to remind herself about her decision to quit (Figure 5).

**Figure 6:**
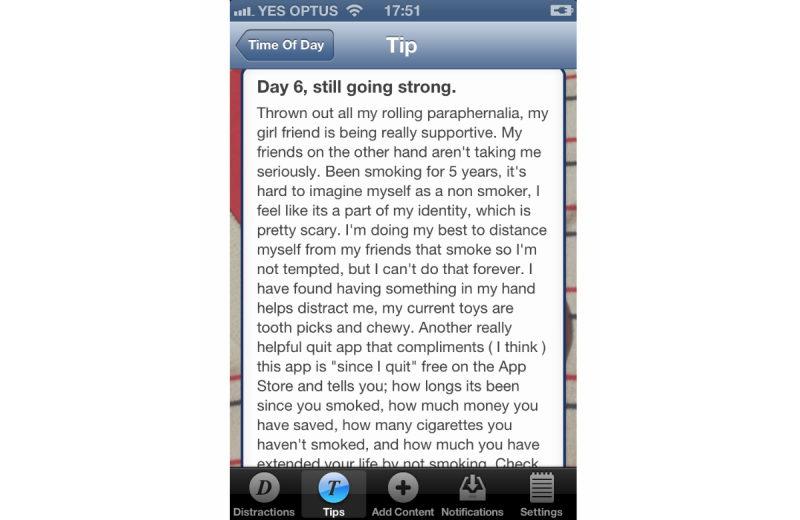
Personal stories written by others who were quitting smoking provided information and reassurance that participants were not alone with their difficulties in quitting.

A key benefit for many participants was to read short personal stories written by others users. As shown in Figure 6, these stories typically provided tips on how to stay quit and they expressed the difficulties of coping with cravings and of resisting temptations to smoke again. These stories were reported as reassuring participants that they were not by themselves with their difficulties, and thereby strengthened their commitment to stay on track.

I really liked [the stories]—I said last time that I didn’t really care about what other people said on the app, but there are stories, like people saying how long it’s been. . . I think I commented on people’s little stories, like when someone hasn’t had a cigarette for 3 days and they were talking about how difficult that was and how good they felt after the first day, which is really true. If you are smoking every day and you can go a day without one, it’s quite a big achievement. And then the second day again, you go through a similar sort of thing. So that helps. It was good to see that those early stages are very similar for those people.

Participant 8, interview 2

Some participants further commented on the benefits of sharing their stories. While the research team seeded the majority of stories, 3 participants shared their quitting stories through the tips section of the app. As indicated by participant 2, being able to share one’s experiences and reading in comments how these posts had helped others to remain nonsmokers, further fortified quitting.

I felt enormously pleased when I saw that people had added some of my tips and things to their favorites – that made me feel really good. I’m thinking that’s a funny response, but it was nice to know that no matter how small I was able to provide someone with something that might have helped them, even if it was only for five minutes.

Participant 2, interview 2

#### Avoidance of High-Risk Situations

Five participants reported that they used the DistractMe app to learn about ways of avoiding situations that may lead to cravings, or they shared their strategies with their peers. Similar to diversion, avoidance seeks to draw attention away from the desire for cigarettes, but does so in an anticipatory way.

A commonly used form of avoidance was to avoid alcohol for the first few weeks after quitting, because consuming alcohol was often associated with meeting other smokers and also because it diminished the control to resist temptations. The following comment on DistractMe is an example of an avoidance strategy shared with peers. Two participants commented on this tip, expressing that they found it useful but also that they were concerned that drinking alcohol again in the future may lead to a relapse.

I’ve given up alcohol for a few weeks as I think it might help me with giving up. Alcohol and cigarettes have formed such a close bond in my head that I think this might be a way to break that bond. In a month I won’t be a smoker anymore so don’t think the alcohol will have the same connection then.

Participant 2, posted on DistractMe

Other participants also highlighted the importance of avoiding particular places and routines. Three participants quit while they were traveling and thereby avoided any reminders through places or routines associated with smoking. Participant 11, on the other hand, temporarily moved in with her boyfriend to avoid temptations associated with her daily routines.

#### Displacement of Smoking With Other Activities

Displacement involves doing something else to prevent cravings. For example,

I kept myself busy doing other stuff that I didn’t even stop to think about needing a distraction.

Participant 1, interview 2

Like avoidance, displacement was an anticipatory strategy that planned to draw attention away from smoking. In contrast to avoidance, however, displacement was about creating new situations in which smoking was not an issue at all. For example, participant 5 posted on DistractMe:

Day one over Grandbaby was a good distraction now I hope I can make it the rest of my life :)

Participant 5, posted on DistractMe

Nine participants mentioned displacement strategies in their posts on the app or in their feedback through the diaries and interviews. As indicated in the post above, spending time with nonsmokers was a common approach. Several people displaced cigarettes with other physical objects like sweets, chewing gums, and drinking straws, and shared these suggestions on the app. A further focus in the displacement activities was on keeping their hands busy through activities like knitting, cleaning, and painting. Several participants saw quitting as part of a larger attempt to develop a healthier lifestyle. They sought to exercise regurlarly, which served as a displacement activity. Beyond that, exercise helped the participants to lift their mood and to cope with other withdrawal symptoms, like feelings of restlessness, stress, and irritability.

Another thing I do to distract myself is I’ve been going to the gym a lot, which is another good benefit, like health benefit, but it’s just also a lot of... it’s an energy outlet as well, because I think I get real tense and a little bit hyperactive, because I’ve got all of this stress going on because I’m not smoking, even though I’m not that stressed, which is weird, I think it’s just internal.

Participant 11, interview 2

## Discussion

### Principal Results

In this study, we have explored how mobile phones might serve as tools to help people remain nonsmokers. Our approach has been to design and evaluate the DistractMe app, which was presented to quitters as a tool to distract themselves by viewing engaging nonsmoking-related content. This design was motivated by reports that self-distraction is a common quitting technique [23]. To provide a comparison in our observations, explicitly smoking-related quitting tips were added to DistractMe. Also included were standard social media functions for sharing comments on items, and viewing visits and favorite ratings. By observing 14 quitters using the DistractMe app, we have attempted to understand how this technological intervention is appropriated into real life quit attempts. Our aim has been to elucidate what forms distraction might take using a mobile app, and to understand this in relation to the use of smoking-related information as provided in the tips and social connectivity to a group of quitters.

Overall, the picture that has emerged through the interviews, diaries, and log data, provides support for the viability of distraction-related techniques in real life quitting using a mobile phone. However, it also reveals highly idiosyncratic use of the DistractMe app. Participants appropriated the app in distinct ways into their particular approach to quitting. We identified 6 quitting strategies deployed in concert with the use of DistractMe. Some of them were intended in the design of the app, whereas others were not. The primary intended strategy of diversion, using distraction items to take the mind off cravings, was reported by just 3 of the participants. In addition, 4 other participants used a strategy of confrontation, by watching or reading tips about smoking to confront cravings as they occurred. Four anticipatory strategies that attempted to prevent cravings or reduce their effects were also reported. Five participants used DistractMe as part of preparation for quitting; this involved reading tips and selecting distractions for later use. Once into the quit stage, 11 of the 14 participants followed a strategy of fortification of the quit attempt; typically this involved reading tips and comments on DistractMe to strengthen their motivations and resolve to remain nonsmokers. The other 2 anticipatory strategies shifted attention off smoking: avoidance strategies focused on staying away from places, people, activities, and situations associated with cigarettes; whereas displacement strategies were more proactively focused on shaping a new, smoke-free lifestyle, often in combination with increased exercise. Here, DistractMe served as a tool for participants to find out and share effective techniques, with 5 reporting valuable exchanges for avoidance and 9 for displacement.

The influence of social exchange between quitters, through posting distractions and tips and through comments on them, pervaded all 6 strategies. This is true even though the volume of exchange was slight relative to many social media applications. In this sense, the DistractMe app provided access to a community of peers that offered various forms of support. They offered informational support to prepare for quitting and to confront cravings. Furthermore, many tips were conveyed through short personal stories that offered emotional support and encouragement to fortify the quit attempt, similar to interactions on discussion forums, blogs, and Facebook pages dedicated to quitting smoking [36-38].

With both distractions and tips, the findings suggest that the main benefit was in consuming them rather than in contributing content. As in most online communities [32], particularly in sensitive settings such as health and behavior change [39], the participants in this study generally wished to remain private and preferred to consume content alone. Nevertheless, all of the 14 participants also contributed content through distractions, tips, and comments on the app. Some participants reported that being able to express themselves fortified their quit attempt, similar to how some quitters use blogs to express themselves and reflect on their progress [40]. More generally, writing one’s story is recognized to have positive effects for health and well-being because it can be cathartic and helps individuals to gain analytical distance from their concerns [41].

While our initial design intention with DistractMe was to support coping through distraction (ie, diversion), the findings showed that the participants more commonly sought to cope with cravings through fortification and confrontation and by paying attention to smoking and cravings, rather than shifting attention away from them. This finding differs from previous research that suggests that distractions are the most frequently applied coping strategy to resist smoking [23]. One limitation pointed out by the participants was that DistractMe did not provide sufficient updates to offer an engaging experience, particularly when compared with the large number of updates on popular social media like YouTube and Facebook. However, the findings also showed that they rarely used these social media to distract themselves either.

A further concern raised by some participants was that the content in DistractMe was mostly not interactive enough and this allowed them to smoke while using the app. Again, however, participants rarely used the highly interactive games that were available on their phones to seek distraction. Overall, we concluded that although the idea of playful diversion through an app appealed to the participants during the initial interviews, they typically did not seek to divert themselves when cravings actually occurred. An equal strategy to diversion was confrontation of the desire, sometimes by using the content of the app to focus on their reason to quit. However, more important than either of these strategies, greater reliance was placed on careful planning for avoidance and displacement. Hence, fortification of the quit attempt was the most widely reported strategy in this group of 14 smokers; chiefly reminding themselves of the negative effects of smoking and by exchanging stories with their peers that encouraged them to stay on track.

Although the findings showed that the DistractMe app offered important resources for immediate coping, the primary benefit for the participants, therefore, was to receive support before cravings even emerged. Typically, they used DistractMe in their downtime, browsing through items to help them prevent cravings, or looking to see if anyone has posted new content. For some participants, looking for new stories and comments on DistractMe became part of their digital routine, like checking emails and other social media. This helped to prepare for risk situations and reduce the risk of relapse at a later point [10,42].

### Limitations

The findings presented here are based primarily on self-report. Although log data were used to verify that each participant presented in this study engaged with the app, it was mainly data from interviews and diaries that informed how DistractMe was used. Influences that occur from automated or operational processes [43] and those most readily forgotten will not be adequately reported. We used diaries to minimize reliance on retrospective accounts of coping from the interviews. Other techniques such as ecological momentary assessment [9] might overcome this limitation, but might also be counterproductive to the aim of distracting users from cigarettes, or might act as an intervention in its own right.

The study was based on a small qualitative study. This allowed us to study only with genuine quit attempts and to examine coping strategies in depth. Although the study allowed us to develop a conception of 6 quitting strategies, it does not offer reliable data on the effectiveness of DistractMe on smoking cessation. Furthermore, the small sample did not generate sufficient content updates for a self-sustaining support community. Relying on the research team to create some of the content introduced some artifice in this respect.

### Recommendations for Practice

The findings of this study suggest several recommendations for the implementation of mobile app interventions to help people stay quit.

The first recommendation is that distractions delivered on a mobile app offer a viable technique for quitters, with the proviso that there are regular updates of content, if necessary, from moderators. Although only a few participants used distractions as a form of diversion from cravings, they provided a compelling reason for quitters to take up the app and explore its potential. Distractions were attractive to some potential users because they framed the app as a lightweight and playful resource to remain nonsmokers. They also facilitated a transition from consuming content to sharing content. Participants found it easier to share distractions than tips because they were easier to find and involved little personal information. This is important, because peer support groups in general relies on contributions from their members to be sustainable in the long term [32], and particularly in areas such as health behavior change many members are reluctant to contribute due to privacy concerns [39].

The second recommendation for smartphone apps to help quitting is to bolster tips conveyed through personal stories because these are attractive to many quitters who are drawn to personal information about the situations of other users. Reading how other people have experienced similar situations and acknowledging that quitting is challenging provided a sense of connectedness and solidarity among some participants that fortified their quit attempts. The high level of anonymity and the ability to comment and engage with stories encouraged some participants to share their experiences, despite concerns about failing to quit.

For an app like this to remain engaging for quitters, it needs to attract a large group of users to develop a self-sustainable online community. A larger group is more likely to generate a variety of distractions and tips, particularly the personal stories that many users desire. Moreover, a larger group is more likely to increase commentary on other people’s posts, which provides recognition and further facilitates the sharing of distractions and tips [32]. This may also help to retain users who successfully quit to share their stories, similar to other online communities in the domain of smoking cessation [37,44].

### Conclusions

This study shows that mobile apps designed around the techniques of distraction and quitting tips can be taken up and used by people attempting to quit smoking. The observations of 14 real life quit attempts revealed that uptake was idiosyncratic and was used as part of 6 distinct quitting strategies: diversion, avoidance, displacement, preparation, fortification, and confrontation. Social exchanges between quitters supported by the app played an important role in the way the app was appropriated. Users shared strategies for long-term distractions to avoid and displace activities, people, and places that may evoke cravings. Quitting tips, on the other hand, helped users to prepare coping strategies and to fortify their quit attempts.

In future work, we plan to expand this work from a small group to a larger community to explore the benefits of self-expression and interactivity among peers, to improve the diversity and depth of distractions and tips available on the application, and to work toward a self-sustainable community where quitters help one another resist smoking. We also plan to see whether there are benefits of integrating the next generation of this app with other online support such as tailored advice and the opportunity to explore the site material on a larger interface such as a tablet or computer screen.

## Multimedia Appendix 1

Questions for interview 1.

## Multimedia Appendix 2

Questions for interview 2.
