# Supplementary material for: A Mobile App Offering Distractions and Tips to Cope With Cigarette Craving: A Qualitative Study
Source: JMIR Mhealth Uhealth. 2014 May 7;2(2):e23. doi: 10.2196/mhealth.3209 (PMC4114415; doi:10.2196/mhealth.3209)
Supplement: Supplementary file 1 [file mhealth_v2i2e23_app1.pdf]

Questions for interview 1

Current status

Could you tell me more about your current status with regards to smoking or quitting?

How many cigarettes do you smoke per day?

Can you talk me through your day and the situations where you smoke?

Last quit attempt

Can you tell me more about your last quit attempt? How did you quit?

What situations did you find challenging? How did you cope with these situations?

Can you tell me how you your quit attempt ended?

Information on how to stay quit

Where do you find information on how to stay quit?

Has anyone around you quit? Can you tell me more about your conversations with him/her?

Have you searched for information about staying quit on the Internet? Can you tell me more about these websites?

Have you tried any of Quit Victoria's services – their website, SMS service, telephone hotline? Can you tell me more about your experiences?

IPhone use

Let's talk about your use of the iPhone more generally – can you show me your three favourite apps?

Where do you use your iPhone? Are there any situations where you cannot access it?

Have you used any apps on your phone to help you stay quit? Can you tell me more about these apps?

Immediate feedback on DistractMe

What is your first impression of the application?

What can we do to improve the app?

What distractions / tips do you find useful?

What distractions/tips will you add to the application?

Explore distractions

What other "distractions" (hobbies, interests) do you have in your life?

What other apps, websites, games etc. keep you distracted?

What do you do when you commute to work / have a break at work / spare time in the evening / on weekends?
