# Supplementary material for: A Mobile App Offering Distractions and Tips to Cope With Cigarette Craving: A Qualitative Study
Source: JMIR Mhealth Uhealth. 2014 May 7;2(2):e23. doi: 10.2196/mhealth.3209 (PMC4114415; doi:10.2196/mhealth.3209)
Supplement: Supplementary file 2 [file mhealth_v2i2e23_app2.pdf]

Questions for interview 2

Current status & quit attempt

What is your current situation: are you quit or are you smoking?

[If smoking] Since we last met, did you make a quit attempt?

Can you talk me through your quit attempt? When did you start your quit attempt? How did you quit? (cold turkey, medication)

Cravings & DistractMe

Can you tell more about situations where you experienced cravings? What triggered those cravings? How often did the cravings occur?

How did you cope with these cravings? Can you give me an example?

What role did the app play to cope with cravings? Can you talk me through an episode?

What was the outcome? How effective was the app to combat cravings?

What content was more useful - tips or distractions? Can you tell me more about this? Can you show me an example?

Other distractions & tips

Did you use any other apps to stay quit? Can you tell me more about them? How do these apps compare to DistractMe / what do they offer?

Where else did you find distraction from cravings? (other apps, TV, hobbies, …)

Can you tell me more about the people supported you in your quit attempt?

What other resources did you use? Where else did you find support to stay quit?

Evaluation of DistractMe

How often did you use the app in general / for such situations?

Can you tell me more about situations where Distract Me didn’t work as expected?

Can you tell me about other situations where you used the app?

Can you show me what content / comments you have added? Can you tell me more about that content / comment?

DistractMe was prototype developed for research, rather than a full-fledged app. What do you think about the principle ideas – distractions, tips, and interactions with other users?

What did you think about the look and feel of DistractMe?

Overall, was DistractMe usable for you – any specific annoyances or delights?

If you could change 3 things to make the app more useful for you, what would they be?
